# Association Between Care Modality and Use With Treatment Response Among Members Accessing Virtual Mental Health Services: Real-world Observational Study

**DOI:** 10.2196/36956

**Published:** 2022-07-22

**Authors:** Emily Shih, Brandon S Aylward, Sarah Kunkle, Grant Graziani

**Affiliations:** 1 Ginger San Francisco, CA United States

**Keywords:** behavioral coaching, engagement, mental health, telehealth, treatment response

## Abstract

**Background:**

There is a growing bottleneck in mental health care, as the demand for services has outpaced the availability of mental health professionals. Consequently, many health systems have shifted to teletherapy as a scalable approach to increasing accessibility to care. Within these care models, various treatment modalities (eg, coaching and clinical care) are used to deliver support for anxiety and depression. However, more research is needed to better understand the differences in treatment responses.

**Objective:**

The purpose of this study was to examine the association between different care modalities and the levels of use with symptom score changes for members seeking virtual care services.

**Methods:**

We conducted an observational study of 4219 members who accessed Ginger, an on-demand mental health service, between September 2020 and September 2021. Using a mobile app, members can access text-based behavioral health coaching and virtual clinical services. This study focused on members with clinically elevated depression or anxiety levels at baseline. Logistic regressions were used to assess the association between care modalities and the levels of use with treatment response in depression and anxiety, using the Patient Health Questionnaire and Generalized Anxiety Disorder Assessment, respectively.

**Results:**

Of the 4219 members, 1623 (38.47%) demonstrated a full response to depression, and 1684 (39.91%) demonstrated a full response to anxiety. Members who completed care (ie, text-based coaching, virtual clinical therapy, hybrid of coaching, and clinical care) beyond the introductory session showed significantly increased odds of a full response compared with those who completed only limited care. Members who completed a hybrid of care had the highest odds of improvement; the odds of showing a full response in depression were 2.31 times higher (95% CI 1.91-2.80; *P*<.001) and in anxiety were 2.23 times higher (95% CI 1.84-2.70; *P*<.001) compared with members who completed limited care. For members who completed only coaching or clinical care, the largest effects were observed among those with high use. For members who completed a hybrid care program, we observed similar treatment responses across all levels of use.

**Conclusions:**

Our real-world study found that members who completed text-based coaching achieved full treatment responses at similar rates compared with members who completed virtual clinical care and members who completed a hybrid of care. There were no significant differences in the predicted probabilities of full treatment response between coaching and clinical care. Generally, the odds for a full response were highest among members with high use within each care modality; however, there were no differences in full-response treatment odds across levels of use with hybrid care. The results support the utility of digital behavioral health interventions and further highlight text-based coaching protocols as an accessible and suitable option when considering virtual care for treating anxiety and depression.

## Introduction

### Background

Before the current pandemic, the prevalence of mental health illness in the United States was more than 1 in 5 adults (20.6%) [1]. Anxiety and depression result in an estimated global economic cost of US $1 trillion each year for lost productivity, absenteeism, and medical costs [2]. The confluence of physical health risks, financial stressors, social isolation, and disruption of daily activities during the COVID-19 pandemic has had a profound impact on the number of individuals with clinical depression and anxiety [3]. In fact, the Centers for Disease Control and Prevention reported that between August 2020 and February 2021, the percentage of adults with recent symptoms of anxiety or depression increased from 36% to 42%, and the percentage of those reporting an unmet mental health care need increased from 9% to 12% [4]. Despite the urgent need for mental health care, individuals still face many barriers in accessing effective treatment services, including high out-of-pocket expenses and transportation challenges [5,6]. In addition, there has been a long-standing care delivery bottleneck for mental health care as the demand for care has outpaced the availability of qualified mental health professionals. Long waitlists have only worsened during the pandemic and underscore the urgency to identify innovative new models to increase access to effective mental health care.

To address the pressing need for services, many health systems and organizations have shifted to teletherapy and other digital interventions as scalable approaches to increasing accessibility to mental health care [7]. Within these care models, various treatment modalities (eg, self-guided content, coaching, and teletherapy) are used to deliver support for anxiety and depression. The efficacy of virtual teletherapy for the treatment of mood and anxiety disorders has been well established, with outcomes similar to face-to-face visits and greater efficacy compared with treatment as usual or placebo [8-11].

Although virtual care can increase access and reach to care, some approaches still rely on the limited supply of highly trained mental health specialists (eg, clinical psychologists). A promising and increasingly popular method of care is behavioral health coaching, which can serve as a lower-intensity alternative to care. Although this type of care may not be suitable for all types of patients (eg, those with suicidal ideation, substance use disorder, or repeated hospitalizations), behavioral health coaching has demonstrated improvements in both the physical and mental health status of patients [12-14]. Care can be delivered by bachelor’s or master’s level providers and represents a more scalable solution that does not overly rely on the limited supply of mental health specialists with advanced training (eg, doctoral degree). Coaching uses methods similar to traditional psychotherapy and can address anxiety and depression through techniques derived from interventions such as mindfulness, solution-oriented focus, and positive psychology [15]. With the rapidly shifting landscape of care models, traditional coaching models have adapted to digital methods, such as text-, video-, or telephone-based coaching. One of the benefits of text-based coaching in particular is that it requires less coaching time and allows for both synchronous and asynchronous support for members, which may be more suitable for the on-the-go lifestyle of those seeking care [16]. Several recent studies using text-based coaching have demonstrated treatment outcomes equivalent to those of in-person and telephone-based care [16-19]. These treatment modalities can serve as scalable solutions to address the growing demand for mental health services; however, more research is needed to better understand the differences in treatment responses for depression and anxiety.

### This Study

The purpose of this study was to examine the association between different care modalities and different levels of use with clinical symptom score changes in members seeking services in a virtual care model. As such, we have two hypotheses: (1) members who completed text-based coaching, clinical sessions, or a hybrid of coaching and clinical care would demonstrate higher odds of a full treatment response (≥50% reduction in symptom scores) for anxiety and depression compared with members who only completed limited care and (2) members who used more sessions would demonstrate higher odds of a full treatment response.

## Methods

### Overview

We conducted a retrospective observational study of members who accessed Ginger, an on-demand mental health service, between September 2020 and September 2021. This is a secondary analysis of pre-existing deidentified data. The study team did not have access to participants’ identifying information and did not intend to recontact the participants.

### Ethics Approval

Ginger’s research protocols and supporting policies were reviewed and approved by Advarra’s institutional review board (Pro00046797) in accordance with the US Department of Health and Human Services regulations at Title 45 Code of Federal Regulations Part 46 [20].

### Participants

Study participants had access to Ginger services as part of their employment or health plan benefits. Internal clinical protocols include exclusionary criteria where self-directed telehealth is likely not appropriate and where more specialized and urgent psychiatric services are required (eg, active suicide ideation and active high-risk self-harm behavior) [21]. This study included Ginger members aged ≥18 years who screened positive on either the Patient Health Questionnaire-9 (PHQ-9) or Generalized Anxiety Disorder-7 (GAD-7) at baseline (ie, score≥10).

### Procedures

The Ginger platform provides members with access to text-based behavioral health coaching, virtual clinical services, and self-guided content and assessments primarily via a mobile app platform. Examples of self-guided content include mindfulness meditation activity cards and stress-management exercises. After downloading the mobile app, members can start texting with a behavioral health coach within minutes of requesting connection. During the coaching sessions, members and coaches work together to set goals and work plans to achieve those goals. Goals can range anywhere, from career goals and relationship goals to other personal goals that the member or coach identifies as a source of anxiety or depression. Members typically begin with text-based coaching sessions and many members remain solely at this level of care. Some members will request clinical care (teletherapy or telepsychiatry), and some will require treatment escalation if the coaches identify a clinical need. Clinical severity was ultimately determined by clinicians using the PHQ-9 and GAD-7, in addition to other assessments that gauge the risk and urgency for clinical care. Examples of situations that require escalation include individuals with chronic mental illness and severe trauma, the potential to harm oneself or others, and significant mental instability (eg, hallucinations, delusions, and extreme mood swings). Members who met certain risk thresholds were advised to escalate to therapy or psychiatry. When members are escalated to therapy or psychiatry, they may continue working with a coach, provided they seek additional specialized care concurrently. Members who did not meet these thresholds were recommended to continue coaching unless they had a specific preference for clinical care.

Ginger coaches are full-time employees who have an advanced degree in a field related to mental health or have accredited coach certification (as approved by the National Board for Health and Wellness Coaching). Coaches are also required to have at least 2 years of relevant experience, of which 6 months must have occurred with direct supervision under a qualified, credentialed, or licensed supervisor. To ensure ongoing quality in the delivery of care, Ginger coaches are trained for at least 200 hours each year on up-to-date effective methodologies (eg, motivational interviewing and goal setting). Ginger clinicians are full-time employees who have completed a minimum master’s degree in psychology, social work, counseling, marriage and family therapy, or a related field. They are licensed to practice (eg, licensed clinical social worker, licensed marriage and family therapist, or licensed psychologist) and undergo quarterly training on protocols, evidence-based care, and best practices in telehealth. Additional details regarding the Ginger care model and providers can be found in previous publications [3,21].

To help providers assist with personalized care, members can track changes in depression and anxiety symptoms during their care journey. Members were prompted to complete the PHQ-9 and GAD-7 through the platform when they began using Ginger services to measure their baseline symptom scores. Symptom surveys were administered every 2 weeks to members who scored above the clinical threshold (≥10) at baseline. The most recent survey response that fell within a 6- to 16-week window following a member’s baseline was considered their follow-up and used for analyses in this study.

### Measures

The PHQ-9 is a 9-item self-report questionnaire based on the Diagnostic and Statistical Manual of Mental Disorders, 4th edition, that assesses the frequency and severity of depression symptoms over the previous 2 weeks [22]. Each of the 9 items is scored on a scale from 0 (not at all) to 3 (nearly every day). Total scores range from 0 to 27, with higher scores indicating more depressive symptoms. A score of 10 is often used as the clinical threshold [22]. Scores of 10 to 14, 15 to 19, and >20 represent moderate, moderately severe, and severe depression, respectively [22]. On the basis of the literature, a reduction in PHQ-9 score of >50% is considered a *full response* [23]. This approach to calculate treatment response better accounts for the variation in baseline severity and, as such, enables a more unbiased measure of symptom improvement.

The GAD-7 is a self-report questionnaire based on the Diagnostic and Statistical Manual of Mental Disorders, 4th edition, diagnostic criteria to assess the frequency and severity of anxious thoughts and behaviors over the past 2 weeks [24]. Each of the 7 items is scored on a scale from 0 (not at all) to 3 (nearly every day), with total scores ranging from 0 to 21. Consistent with the existing literature, a score of 10 was used as the clinical threshold for this study [25]. Scores of 10 to 14 and >15 represent moderate and severe anxiety, respectively [24]. Similar to the threshold used for the PHQ-9, a reduction in the GAD-7 score of >50% was considered a *full response* to treatment.

### Care Modality and Levels of Use

We calculated the use based on product user behavior data. Coaching sessions were operationalized as the number of unique days on which members and coaches exchanged at least five text messages. We decided on this threshold because we learned from the internal provider feedback that the first few messages are generally characterized as introductory and administrative-related messages with minimal therapeutic intervention. By definition, it is not possible to have more than one coach session per day. Clinical sessions were operationalized as the number of completed video sessions with a clinician and scheduled on a need basis. Each video session was typically an hour long. We further categorized use based on member use of different platform care modalities (ie, text-based coaching and clinical sessions) and the number of times they used these modalities (ie, minimum, low, moderate, and high). This study considered 4 different care modalities. Members who did not complete any coaching or clinical sessions and members who only completed one coaching or one clinical session or one of both were categorized in the *Limited Care* cohort. We included members who completed one coaching or one clinical session or one of both in the *Limited Care* cohort, because, generally, these first sessions are considered introductory, where providers introduce themselves and delineate the structure and plan for the member’s care journey. Members who completed more than one text-based coaching session and either none or one clinical session were categorized as the *Coaching Only* cohort. Members who completed more than one clinical teletherapy session and either none or one coaching session were categorized as the *Clinical Only* cohort. Finally, members who completed more than one coaching and more than one clinical session were categorized as the *Hybrid Care* cohort.

Previous literature has not yet established an optimal threshold for on-demand text-based care. As such, we adopted a data-driven approach to operationalize the levels of use. Different levels of use were determined for the care modalities, Coaching Only, Clinical Only, and Hybrid Care, based on quartiles of completed sessions within each care modality cohort (25th, 50th, and 75th percentiles). Members who fell below the 25th percentile for their respective cohort were categorized as *Minimal Use*. Members who fell between the 25th percentile and the 50th percentile median were categorized as *Low Use.* Members who fell between the 50th and 75th percentiles were categorized as *Moderate Use* and members who scored above the 75th percentile were categorized as *High Use*. The exact number of sessions for each group is shown in Table 1.

**Table 1:** Description of levels of use by care modality cohort (N=4219; Limited Care n=1072).

Level of use		Definition by quarter percentiles, sessions	Participants, n (%)
**Coaching Only (n=1354)**			
	Minimal	≤2	329 (7.79)
	Low	3 to 4	345 (8.18)
	Moderate	5 to 7	332 (7.86)
	High	≥8	348 (8.25)
**Clinical Only (n=941)**			
	Minimal	≤3	363 (8.6)
	Low	4	169 (4.01)
	Moderate	5 to 6	212 (5.03)
	High	≥7	197 (4.67)
**Hybrid Care (n=852)**			
	Minimal	≤7	260 (6.16)
	Low	8 to 9	173 (4.1)
	Moderate	10 to 13	231 (5.48)
	High	≥14	188 (4.46)

### Data Management and Analysis

Analyses were conducted using R Studio (version 1.4.1717; RStudio). Data were first screened for outliers and normality. Separate ANOVA models were used to evaluate whether members varied in their baseline depression and anxiety scores. Given the binary nature of our dependent outcome variable (*full response* vs *no full response*), we used logistic regression modeling, a common statistical method for quantifying the relationship between various factors and a binary clinical outcome. In our first set of analyses, 2 binary logistic regression models were used to explore the association between care modality and the likelihood of demonstrating a full response in depressive and anxiety symptoms. These logistic regression models produce estimates of the probability of demonstrating reductions in depressive and anxiety symptoms when a member is in a particular group that represents a modality of care. The likelihood of each modality group demonstrating a full response relative to the reference group is shown by odds ratios. In addition, 6 logistic regression models were used to explore the association between levels of use within each care modality and treatment response for depression and anxiety symptoms. Members’ depression and anxiety symptom scores at baseline were included as covariates in all models to account for variations in the baseline symptom scores. The performance of the logistic regression models was evaluated using the Hosmer-Lemeshow test to measure model fit, and odds ratios and 95% CIs were calculated to show associations with improvements in depression and anxiety. The models were constructed using complete case analyses.

## Results

### Participant Demographics and Characteristics

A total of 6466 members participated in this study. Of those 6466 members, 4219 (65.25%) screened positive for depression or anxiety (ie, PHQ-9 or GAD-7 symptom scores ≥10). This study focused only on members who screened positive at baseline (N=4219), which will be subsequently referred to as the analytical sample. The complete descriptive statistics are reported in Table 2. Of the 4219 members in the analytical sample, 1645 (38.99%) members were identified as female, 689 (16.33%) as male, 142 (3.37%) as other, and 1743 (41.31%) did not have gender identity information available. Of the 4219 members, a total of 1613 (38.23%) members were aged <35 years, 1264 (29.96%) members were ≥35 years, and 1342 (31.81%) members did not have their age reported.

**Table 2:** Characteristics of the analytical sample (N=4219)^a,b^.

Characteristics		Analytical sample
**Age (years), n (%)**		
	18 to 34	1613 (38.23)
	>35	1264 (29.96)
	No response	1342 (31.81)
**Gender, n (%)**		
	Female	1645 (38.99)
	Male	689 (16.33)
	Other	142 (3.37)
	No response	1743 (41.31)
**Care modality, n (%)**		
	Limited Care	1072 (25.41)
	Coaching Only	1354 (32.09)
	Clinical Only	941 (22.3)
	Hybrid Care	852 (20.19)
GAD-7^c^ baseline score, mean (SD)		12.7 (4.54)
PHQ-9^d^ baseline score, mean (SD)		13.7 (5.04)

^a^Counts and percentages were reported for categorical variables.

^b^Mean and SDs were reported for continuous variables.

^c^GAD-7: Generalized Anxiety Disorder-7.

^d^PHQ-9: Patient Health Questionnaire-9.

On average, members completed 3.53 (SD 3.81) text-based coaching sessions and 2.32 (SD 2.85) clinical sessions. Of the total number of members in the analytical sample, 25.41% (1072/4219) received limited care, 32.09% (1354/4219) completed only text-based coaching, 22.3% (941/4219) completed only clinical care, and 20.19% (852/4219) completed a hybrid of coaching and clinical care.

The average baseline scores on the PHQ-9 and GAD-7 were 13.7 (SD 5.04) and 12.7 (SD 4.54), respectively. A total of 75.07% (3167/4219) of the participants demonstrated at least one unit of improvement in their PHQ-9 score from baseline to follow-up, with an average unit decrease in symptom scores of 4.43 units (SD 6.27). On the GAD-7, 72.05% (3040/4219) of members demonstrated at least one unit of improvement in anxiety scores from baseline to follow-up and the average unit decrease in scores was 4.08 (SD 5.82). With regard to treatment response, 38.47% (1623/4219) demonstrated a full response on the PHQ-9 and 39.91% (1684/4219) demonstrated a full response on the GAD-7.

Separate ANOVA models were used to evaluate the differences in PHQ-9 and GAD-7 baseline scores between the care modality cohorts. The results revealed a significant difference between cohorts for both baseline PHQ-9 scores (*F*_3,4215_=12.5; *P*<.001) and baseline GAD-7 scores (*F*_3,4215_=2.99; *P*=.03). Tukey post hoc tests revealed that members who completed a hybrid of care had significantly higher baseline PHQ-9 scores than those who completed limited care (Mean_Hybrid_=14.5, Mean_Limited_=13.5; *P<*.001). Members who completed only text-based coaching sessions had significantly lower baseline PHQ-9 scores than those who completed only clinical sessions (Mean_Coaching_=13.2, Mean_Clinical_=14.0; *P*<.001) or members who completed a hybrid of care (Mean_Hybrid_=14.5; *P*<.001). In addition, members who completed only coaching sessions had significantly lower baseline GAD-7 scores than those who completed only clinical sessions (Mean_Coaching_=12.5, Mean_Clinical_=12.8; *P*=.02). These differences were expected because of the nature of our triaging system used to direct members to the correct level of care. It is also important to point out that the magnitude of these mean differences is small (≤1 point; Table 3). However, owing to significant differences in baseline scores, as indicated by *F* tests, we included both PHQ-9 and GAD-7 baseline scores as covariates in all regression models.

**Table 3:** Descriptive statistics for the Patient Health Questionnaire-9 (PHQ-9) and Generalized Anxiety Disorder-9 (GAD-7) scores at baseline and follow-up, by care modality (N=4219).

	*F* statistic (*df*)	*P* value	Limited care (n=1072), mean (SD)	Coaching Only (n=1354), mean (SD)	Clinical Only (n=941), mean (SD)	Hybrid Care (n=852), mean (SD)
Baseline PHQ-9	12.51 (3,4215)	<.001	13.5 (4.89)	13.2 (4.97)	14.0 (5.14)	14.5 (5.14)
Follow-up PHQ-9	33.5 (3,4215)	<.001	10.8 (6.26)	9.25 (6.28)	8.54 (5.31)	8.39 (5.52)
Baseline GAD-7	2.99 (3,4215)	.03	12.7 (4.61)	12.5 (4.43)	13.0 (4.48)	12.8 (4.65)
Follow-up GAD-7	39.2 (3,4215)	<.001	10.1 (5.84)	8.56 (5.71)	7.97 (5.10)	7.67 (5.37)

### Depression: Full Response by Care Modality

**Figure 1:**
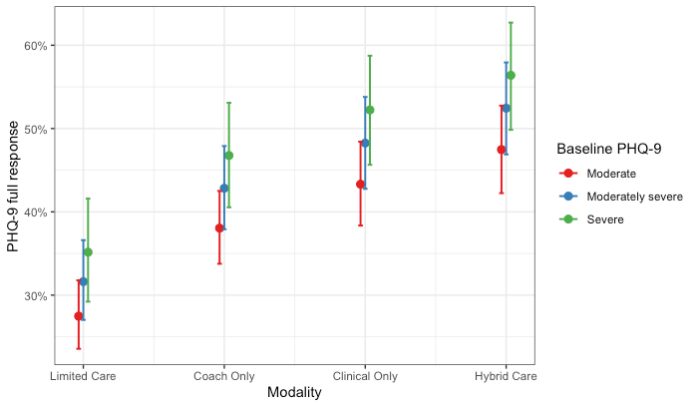
Probability of a full treatment response for depression by care modality and level of baseline severity. PHQ-9: Patient Health Questionnaire-9.

We used a logistic regression model to investigate the association between care modality and full response in depression symptom scores. A Hosmer-Lemeshow test failed to reject the null hypothesis, indicating goodness of fit (χ^2^_8_=6.8; *P*=.56). All modalities showed increased odds of symptom improvement compared with members who completed limited care, but the strongest odds were observed for members who engaged with a hybrid of coaching and clinical care; the odds of showing a full response were 2.31 times higher (95% CI 1.91-2.80; *P*<.001) for those who engaged with hybrid care compared with members who completed limited care. Of note, overlapping CIs among Coaching Only (95% CI 1.41-1.99), Clinical Only (95% CI 1.64-2.38), and Hybrid Care (95% CI 1.91-2.79) cohorts suggest that members in these groups did not differ significantly from one another when predicting depression symptom improvement. Full model coefficients are presented in Table 4. These results are further shown graphically as the predicted probability of a full response in depression scores by care modality and levels of baseline severity (Figure 1).

**Table 4:** Coefficients from the logistic regression model predicting a full response in depression (Patient Health Questionnaire-9 [PHQ-9]) and a full response in anxiety (Generalized Anxiety Disorder-7 [GAD-7]) across care modalities^a^, while controlling for scores at baseline (N=4219).

			Odds ratio (95% CI)		Percentage change (%)		*P* value
**Dependent variable:^b^ full response in PHQ-9**							
	Intercept	0.29 (0.23-0.37)		−243		<.001	
	Coaching Only	1.67 (1.41-1.99)		67		<.001	
	Clinical Only	1.98 (1.64-2.38)		97		<.001	
	Hybrid Care	2.31 (1.91-2.80)		131		<.001	
	PHQ-9 score at baseline	1.05 (1.03-1.06)		5		<.001	
	GAD-7 score at baseline	0.97 (0.96-0.99)		−3		<.001	
**Dependent variable:^b^ full response in GAD-7**							
	Intercept	0.38 (0.29-0.48)		−166		<.001	
	Coaching Only	1.69 (1.42-2.01)		69		<.001	
	Clinical Only	2.20 (1.83-2.66)		120		<.001	
	Hybrid Care	2.23 (1.84-2.70)		123		<.001	
	PHQ-9 score at baseline	0.95 (0.94-0.96)		−5		<.001	
	GAD-7 score at baseline	1.06 (1.05-1.08)		6		<.001	

^a^Reference group for care modality: limited care.

^b^Dependent variable: coded full response=1; no full response=0.

### Anxiety: Full Response by Care Modality

**Figure 2:**
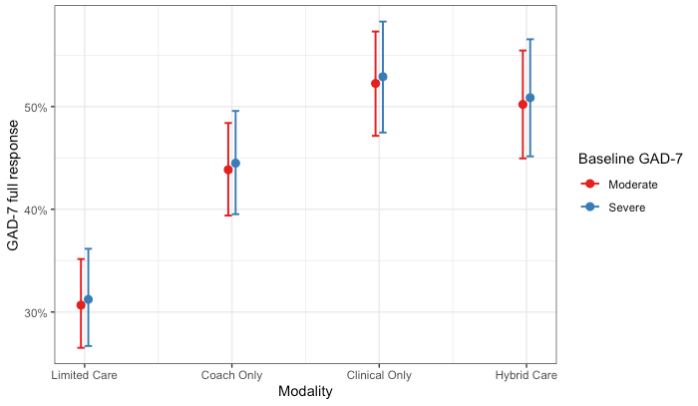
Probability of a full treatment response for anxiety by care modality and level of baseline severity. GAD-7: Generalized Anxiety Disorder-7.

We used another logistic regression model to investigate the association between care modality and full response in anxiety symptom scores. A Hosmer-Lemeshow test failed to reject the null hypothesis, indicating goodness of fit (χ^2^_8_=4.4; *P*=.82). All care modalities showed increased odds of symptom improvement compared with limited care, but the strongest odds were observed for members who completed a hybrid of coaching and clinical care; the odds of showing a full response in anxiety symptom scores were 2.23 times higher (95% CI 1.84-2.70; *P*<.001) for members who completed a hybrid of both clinical and coaching care compared with members who completed limited care. Of note, overlapping CIs among Coaching Only (95% CI 1.42-2.01), Clinical Only (95% CI 1.83-2.66), and Hybrid Care (95% CI 1.84-2.70) cohorts suggest that members in these groups did not differ significantly from one another when predicting full treatment response in anxiety symptoms. Full model coefficients are presented in Table 4. These results are shown graphically as the predicted probability of a full response in anxiety by care modality and levels of baseline severity (Figure 2).

### Coaching Only Cohort: Full Response by Levels of Use

**Figure 3:**
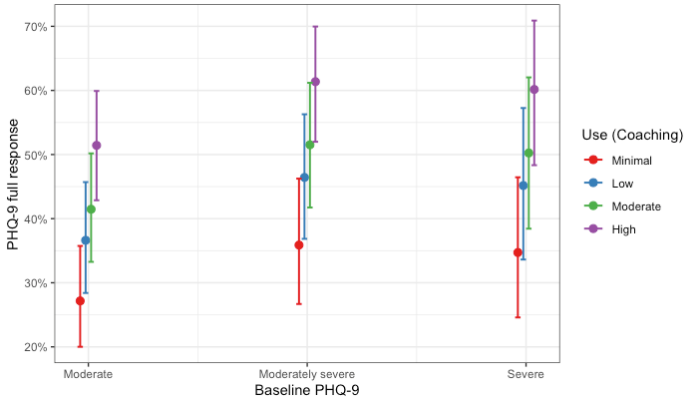
Probability of a full treatment response for depression by levels of use and level of baseline severity for members in the Coaching Only cohort. PHQ-9: Patient Health Questionnaire-9.

**Figure 4:**
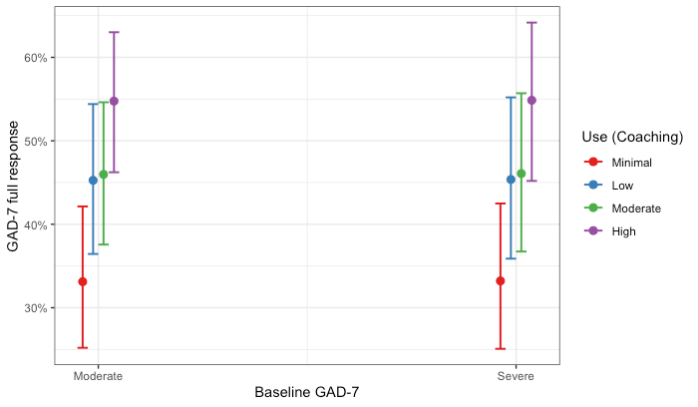
Probability of a full treatment response for anxiety by levels of use and level of baseline severity for members in the Coaching Only cohort. GAD-7: Generalized Anxiety Disorder-7.

Members in the Coaching Only cohort completed, on average, 5.53 (SD 3.65) coaching sessions. Within this modality, we estimated two logistic regression models examining (1) the association between levels of use of text-based coaching and a full response on the PHQ-9 and (2) the association between levels of use of text-based coaching and a full response on the GAD-7. Hosmer-Lemeshow tests failed to reject the null hypothesis, indicating goodness of fit (PHQ-9: χ^2^_8_=9.7; *P*=.29 and GAD-7: χ^2^_8_=5.9; *P*=.66). Full coefficients for both models are presented in Table 5. Compared with members with minimal use, members with moderate and high levels of use had significantly increased odds of treatment response in depression. Specifically, the odds of showing a full response in depression were 2.44 times higher (95% CI 1.77-3.37; *P*<.001) for members with high use compared with members with minimal use. Similar patterns were observed for the model predicting a full response in anxiety. Compared with members with minimal use, all other levels of use had significantly increased odds of treatment response for anxiety. The odds of showing a full response in anxiety were 1.99 times higher (95% CI 1.44-2.74; *P*<.001) for members with high use compared with members with minimal use. The association remained after adjusting for baseline anxiety and depression symptom scores. These results are shown graphically as the predicted probability of a full response by coaching use and baseline severity in Figure 3 and Figure 4 for depression and anxiety, respectively.

**Table 5:** Coefficients from the logistic regression model with the Coaching Only cohort predicting a full response in depression (Patient Health Questionnaire-9 [PHQ-9]) and a full response in anxiety (Generalized Anxiety Disorder-7 [GAD-7]) across levels of use^a^, while controlling for scores at baseline (N=1354).

			Odds ratio (95% CI)		Percentage change (%)		*P* value
**Dependent variable:^b^ full response in PHQ-9**							
	Intercept	0.39 (0.29-0.67)		−159		<.001	
	Low use	1.40 (0.95-1.75)		40		.04	
	Moderate use	1.63 (1.05-1.93)		63		.003	
	High use	2.44 (1.91-3.53)		144		<.001	
	PHQ-9 score at baseline	1.04 (1.02-1.07)		4		.001	
	GAD-7 score at baseline	0.96 (0.93-0.99)		−4		.003	
**Dependent variable:^b^ full response in GAD-7**							
	Intercept	0.46 (0.29-0.72)		−118		<.001	
	Low use	1.48 (1.08-2.05)		48		.02	
	Moderate use	1.84 (1.33-2.55)		84		<.001	
	High use	1.99 (1.44-2.74)		99		<.001	
	PHQ-9 score at baseline	0.94 (0.92-0.96)		−7		<.001	
	GAD-7 score at baseline	1.07 (1.04-1.10)		7		<.001	

^a^Reference group for use: minimal use.

^b^Dependent variable coded full response=1; no full response=0.

### Clinical Only Cohort: Full Response by Levels of Use

**Figure 5:**
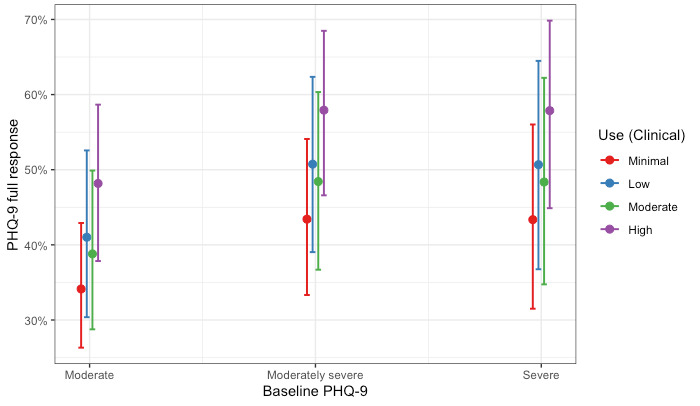
Probability of a full treatment response for depression by levels of use and level of baseline severity for members in the Clinical Only cohort. PHQ-9: Patient Health Questionnaire-9.

**Figure 6:**
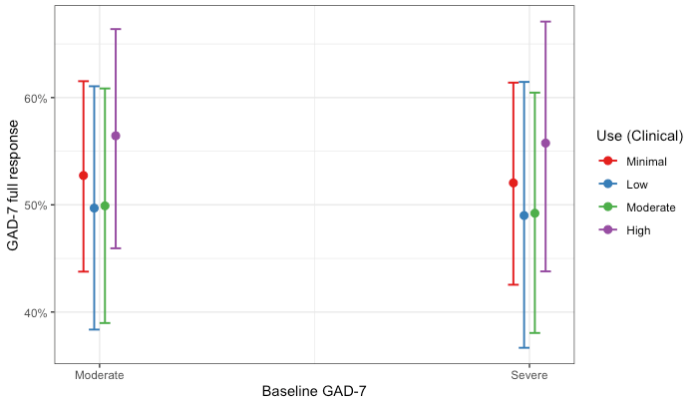
Probability of a full treatment response for anxiety by levels of use and level of baseline severity for members in the Clinical Only cohort. GAD-7: Generalized Anxiety Disorder-7.

The Clinical Only cohort of members completed, on average, 4.69 (SD 2.43) clinical sessions. We estimated two logistic regression models examining (1) the association between levels of use in clinical care and full response on the PHQ-9 and (2) the association between levels of use in clinical care and full response on the GAD-7. Hosmer-Lemeshow tests failed to reject the null hypothesis, indicating goodness of fit (PHQ-9: χ^2^_8_=4.4; *P*=.82 and GAD-7: χ^2^_8_=6.3; *P*=.62). Full coefficients for both models are presented in Table 6. Compared with members with minimal use, all other levels of use had significantly increased odds of treatment response for depression. The odds of showing a full response in depression were 2.06 times higher (95% CI 1.44-2.95; *P*<.001) for members with high use compared with members with minimal use. Different patterns were observed for the model predicting a full response in anxiety. Compared with members with minimal use, only members who had high levels of use had significantly increased odds of treatment response for anxiety. The odds of showing a full response in anxiety were 1.43 times higher (95% CI 1.00-2.04; *P*<.001) for members with high use compared with members with minimal use. The association remained after adjusting for baseline depression and anxiety scores. These results are shown graphically as the probability of a full response by clinical use and baseline severity in Figure 5 and Figure 6 for depression and anxiety, respectively.

**Table 6:** Coefficients from the logistic regression model with the Clinical Only cohort predicting a full response in depression (Patient Health Questionnaire-9 [PHQ-9]) and a full response in anxiety (Generalized Anxiety Disorder-7 [GAD-7]) across levels of use^a^, while controlling for scores at baseline (N=941).

		Odds ratio (95% CI)	Percentage change (%)	*P* value
**Dependent variable:^b^ full response in PHQ-9**				
	Intercept	0.29 (0.17-0.47)	−250	<.001
	Low use	1.84 (1.26-2.68)	84	.001
	Moderate use	1.61 (1.13-2.28)	61	.008
	High use	2.06 (1.44-2.95)	106	<.001
	PHQ-9 score at baseline	1.06 (1.03-1.09)	−2	<.001
	GAD-7 score at baseline	0.98 (0.95-1.01)	6	.26
**Dependent variable:^b^ full response in GAD-7**				
	Intercept	0.53 (0.32-0.87)	−90	.01
	Low use	1.27 (0.87-1.84)	27	.21
	Moderate use	1.25 (0.88-1.76)	25	.21
	High use	1.43 (1.00-2.04)	43	.05
	PHQ-9 score at baseline	0.96 (0.93-0.98)	−4	.002
	GAD-7 score at baseline	1.07 (1.04-1.11)	7	<.001

^a^Reference group for use: minimal use.

^b^Dependent variable coded full response=1; no full response=0.

### Hybrid Care (Coaching+Clinical) Cohort: Full Response by Levels of Use

**Figure 7:**
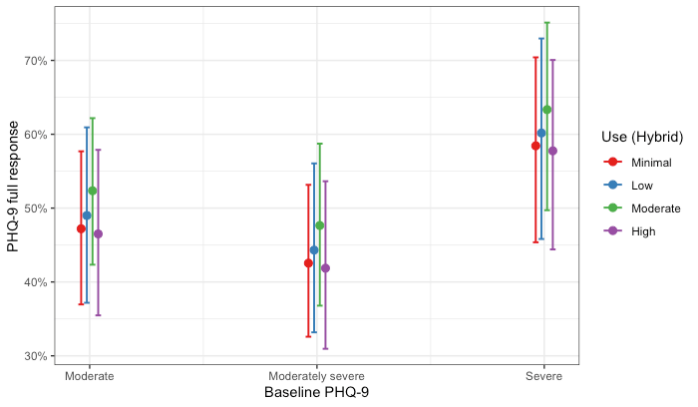
Probability of a full treatment response for depression by levels of use and level of baseline severity for members in the Hybrid Care cohort. PHQ-9: Patient Health Questionnaire-9.

**Figure 8:**
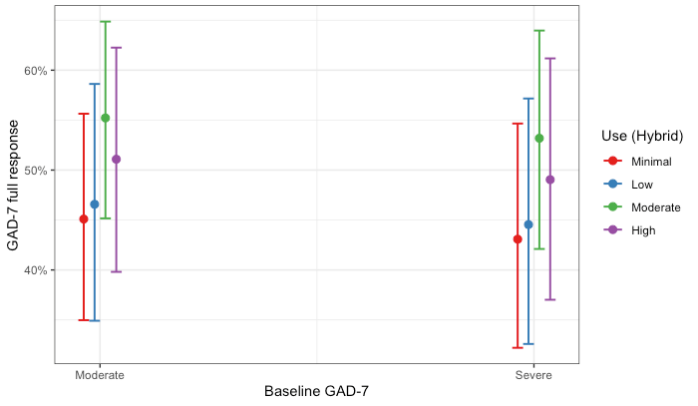
Probability of a full treatment response for anxiety by levels of use and level of baseline severity for members in the Hybrid Care cohort. GAD-7: Generalized Anxiety Disorder-7.

The Hybrid Care cohort of members completed, on average, 10.5 sessions (5.70 coaching sessions; 4.79 clinical sessions). We estimated two logistic regression models examining (1) the association between levels of use and full response on the PHQ-9 and (2) the association between levels of use and full response on the GAD-7 for members who completed a hybrid of coaching and clinical care. Hosmer-Lemeshow tests failed to reject the null hypothesis, indicating goodness of fit (PHQ-9: χ^2^_8_=5.6; *P*=.70 and GAD-7: χ^2^_8_=6.1; *P*=.64). Full coefficients for the models are presented in Table 7. Members with minimal use had similar odds of improvement compared with all other levels of use for both depression and anxiety. The association remained after adjusting for depression and anxiety baseline scores. These results are shown graphically as the probability of a full response by Hybrid Care use and baseline severity in Figure 7 and Figure 8 for depression and anxiety, respectively.

**Table 7:** Coefficients from the logistic regression model with the Hybrid Care cohort predicting a full response in depression (Patient Health Questionnaire-9 [PHQ-9]) and a full response in anxiety (Generalized Anxiety Disorder-7 [GAD-7]) across levels of use^a^, while controlling for scores at baseline (N=852).

			Odds ratio (95% CI)		Percentage change (%)		*P* value
**Dependent variable:^b^ full response in PHQ-9**							
	Intercept	0.61 (0.37-1.03)		−63		.06	
	Low use	1.01 (0.69-1.49)		1		.96	
	Moderate use	1.07 (0.75-1.53)		7		.72	
	High use	0.86 (0.59-1.25)		−17		.43	
	PHQ-9 score at baseline	1.05 (1.02-1.08)		5		.003	
	GAD-7 score at baseline	0.98 (0.95-1.01)		−2		.23	
**Dependent variable:^b^ full response in GAD-7**							
	Intercept	0.61 (0.36-1.02)		−65		.06	
	Low use	1.23 (0.83-1.82)		23		.30	
	Moderate use	1.27 (0.89-1.83)		27		.19	
	High use	1.28 (0.87-1.88)		28		.21	
	PHQ-9 score at baseline	0.96 (0.93-0.98)		−5		.002	
	GAD-7 score at baseline	1.07 (1.03-1.10)		7		<.001	

^a^Reference group for use: minimal use.

^b^Dependent variable coded full response=1; no full response=0.

## Discussion

### Principal Findings

This study examined differences in depression and anxiety treatment response by care modality (ie, limited care, text-based Coaching Only, Clinical Only, and Hybrid Care), as well as by levels of use within each care modality (ie, minimal, low, moderate, and high) in members with moderate to severe baseline anxiety or depression. Overall, nearly 2 out of 5 members demonstrated a full response on the PHQ-9 (1623/4219, 38.47%) and GAD-7 (1684/4219, 39.91%). Our logistic regression models examining the association between care modalities and treatment response found significantly greater odds of full-treatment response in depression and anxiety for members who completed text-based coaching, clinical care, and hybrid care compared with members who completed limited care. The probability of treatment response did not differ significantly between text-based coaching, clinical care, and hybrid care, as indexed by the overlapping 95% CIs, with all 3 modalities estimating a probability of response >35%. In addition to differences by care modality, we also examined treatment responses by levels of use within each care modality cohort. Our data suggest that although all care modalities (ie, Coaching Only, Clinical Only, and Hybrid Care) appeared to offer comparable benefits in managing depression and anxiety above limited care, the treatment responses within each care modality cohort differed among levels of use. The possible explanations for these findings are discussed further.

Members who completed limited care had significantly lower odds of demonstrating a full response on both the PHQ-9 and GAD-7. Given that our analytical sample included only members with a baseline score on the GAD-7 or PHQ-9 ≥10, the established threshold for clinical severity, members likely needed more than limited care (0-1 coaching, 0-1 clinical sessions) to reduce their symptomatology. Members in the Limited Care cohort did not sufficiently interact with a provider, but some could have used self-guided content, although the latter was not explicitly investigated in this study. Our results suggest that interaction with a provider, above and beyond the initial introduction session, offers added benefits in addressing depression and anxiety symptoms. A systematic review of internet interventions for depression found a linear effect on the role of clinician contact, such that between-group Cohen *d* effect size was 0.21, if there was no contact with a clinician either before or during treatment, an effect size of Cohen *d*=0.58 if there was contact with a clinician during treatment, and an effect size of Cohen *d*=0.76 if there was therapist contact before and during treatment [26]. Trends from this study further highlight the importance of offering convenient and accessible telehealth care. In addition, the Ginger model is based on a continuum of care that provides seamless integration and escalation across different levels of care. As such, our findings offer early evidence that members are being properly directed to the correct type of care based on their mental health needs and goals. However, this is speculative and outside the scope of this study; therefore, additional research is required.

We observed similar outcomes in those who completed text-based coaching sessions and clinical care. This result supports prior work suggesting that coaching can be a suitable option for addressing anxiety and depression [12-15], which supplements the literature by investigating text messaging as a viable delivery medium for coaching. The principles of text-based coaching are consistent with how individuals engage with text messaging in their daily routines, and on-demand text-based coaching offers added convenience in terms of not requiring scheduled appointments [16]. Given the scalability of text-based coaching, in which coaches can have simultaneous conversations, this type of care may be a more cost-effective alternative to traditional psychotherapy.

When focusing on the levels of use within care modality cohorts, our results revealed nuanced patterns. For members who completed only coaching or clinical care, the largest effects were observed among those with high use (above the 75th percentile), estimating a probability of response of >45% for both depression and anxiety. Interestingly, for members who completed a hybrid of coaching and clinical care, we saw similar treatment responses across all levels of use (ie, minimal, low, moderate, and high use), with all levels estimating a probability of response similar to the probability of response for members with high use in the Coaching Only and Clinical Only cohorts (approximately 45% for depression and anxiety). Taken together, our results suggest that typically higher use with care would yield better outcomes; however, this was not true for the members in our Hybrid Care cohort, where all levels of use had similar odds of a full treatment response for both depression and anxiety. This is likely owing to the members of the Hybrid Care cohort that used more sessions. On average, members in the Hybrid Care cohort (mean 10.5) completed approximately twice the number of sessions than members in either the Clinical Only cohort (mean 4.69 sessions) or in the Coaching Only cohort (mean 5.53 sessions) within the study period. In addition, members with minimal use in the Hybrid Care cohort completed a similar number of sessions compared with members with high use in the Coaching Only and Clinical Only cohorts. However, the use of more sessions within the Hybrid Care cohort did not demonstrate increased odds of a full treatment response. These findings suggest that more sessions do not always yield better treatment responses, and future research evaluating care should consider nonlinear patterns.

In addition, across all care modality cohorts, the odds of demonstrating a full response were higher for depression than for anxiety. We have considered a couple of reasons to explain these patterns: (1) the Ginger care model demonstrates more support for treating depression within the study time frame than for treating anxiety and (2) the study time frame we assessed (6-16 weeks following baseline) might be a sufficient amount of time to address depressive symptoms, but more time is needed to address anxiety symptoms. These explanations are speculative, and additional research is needed to specifically test these hypotheses.

### Limitations and Future Studies

This study has several limitations. The data set used for this study was limited to people who had access to Ginger services, who had completed the PHQ-9 and GAD-7 survey measures, and whose baseline scores were ≥10. As such, the results are not generalizable to those who do not have access to the system or to those who may have discontinued treatment. A large percentage of members did not have age (1342/4219, 31.81%) or gender (1743/4219, 41.31%) reported, and we also had limited access to other demographic information due to lack of reporting by employers (eg, race and ethnicity, socioeconomic status, and previous mental health treatment). Thus, we were not able to examine the association between these factors and treatment response or stratify the analyses by age or gender. These additional analyses can provide valuable insights into those who may best benefit from virtual care. However, because the Ginger platform is offered through employers, we know that the survey respondents are working-age adults, suggesting that these findings may be generalized to the professional workforce and those enrolled in health benefits through their employer. In addition, we acknowledge that some members could be taking medication, which might affect their responses to care. We do not currently have medication use reported by our members. Thus, our results cannot conclude that the responses to care were not driven by the medication used in combination with therapy. Finally, because this was a retrospective observational study, we lacked a control group to infer the causality of the levels of engagement in treatment response.

The findings of this study generate additional research topics for future studies. Given that the aim of this study was to examine the impact of provider care, we did not specifically evaluate how different levels of use of our self-guided content would impact outcomes. Future research should focus on the impact of self-guided content independent of provider care. In addition, text-based coaching protocols can vary significantly, and some use highly templated messages that facilitate a more efficient coach workflow [16]. A content analysis of coaching messages could provide additional insights into the underlying mechanisms driving symptom improvement in on-demand care.

### Conclusions

Our real-world observational study found that members who completed text-based coaching achieved full treatment responses at similar rates compared with members who completed clinical care and members who completed a hybrid of coaching and clinical care. There were no significant differences in the predicted probabilities of a full treatment response for both anxiety and depression between text-based coaching and clinical care. The highest level of use within each care modality cohort generally had increased odds of treatment response compared with minimal use, with the exception of the Hybrid Care cohort. The results support the utility of digital behavioral health interventions and further highlight text-based coaching protocols as an accessible and suitable option when considering virtual care for treating anxiety and depressive symptoms. Future studies should investigate optimal levels of use.

## Abbreviations

GAD-7: Generalized Anxiety Disorder-7
PHQ-9: Patient Health Questionnaire-9
